# Regulatory T Cell Subsets in Filarial Infection and Their Function

**DOI:** 10.3389/fimmu.2013.00305

**Published:** 2013-09-30

**Authors:** Simon Metenou, Thomas B. Nutman

**Affiliations:** ^1^Helminth Immunology Section, Laboratory of Parasitic Diseases, National Institutes of Health, Bethesda, MD, USA

**Keywords:** tTregs, pTregs, Tr1, Th3, filarial infection, *O. volvulus*, *W. bancrofti*, *B. malayi*

## Abstract

Filarial infections in humans are chronic infections that cause significant morbidity. The chronic nature of these infections with continuous antigen release is associated with a parasite-specific T cell hypo-responsiveness that may over time also affect the immune responses to bystander antigens. Previous studies have shown the filarial parasite antigen-specific T cells hypo-responsiveness is mediated by regulatory cytokines – IL-10 and TGF-β in particular. Recent studies have suggested that the modulated/regulated T cell responses associated with patent filarial infection may reflect an expansion of regulatory T cells (Tregs) that include both Tregs induced in peripheral circulation or pTregs and the thymus-derived Tregs or tTregs. Although much is known about the phenotype of these regulatory populations, the mechanisms underlying their expansion and their mode of action in filarial and other infections remain unclear. Nevertheless there are data to suggest that while many of these regulatory cells are activated in an antigen-specific manner the ensuing effectors of this activation are relatively non-specific and may affect a broad range of immune cells. This review will focus on the subsets and function of regulatory T cells in filarial infection.

## Introduction

### Background

Among the eight filarial humans, four – *Wuchereria bancrofti, Brugia malayi, Onchocerca volvulus*, and *Loa loa* – are considered to be the most pathogenic. These vector-borne parasites cause chronic helminth infections that have infected approximately 200 million people in the tropical and subtropical regions of the world (1–5). In endemic areas, epidemiological studies have grouped people into three major categories based on the presence of parasites and/or the presentation of clinical symptoms. These include: (1) endemic normal (or putatively immune) individuals who, despite chronic exposure to the infectious agents, appear to have no signs of infection and/or pathology; (2) those with pathology or obvious clinical symptoms (e.g., lymphedema in lymphatic filariasis (LF), ocular, or skin disease in onchocercosis, Calabar swelling in loiasis); and (3) those with subclinical infection who often have circulating microfilariae or parasite antigen. It is thought that each of these varying clinical outcomes reflects to some extent the nature of the immune (regulatory or inflammatory) response (6–12). Moreover, these asymptomatic individuals are known to have a diminished parasite-specific CD4+ proliferative and cytokine (particularly IL-2, IFN-γ) responses; with longstanding infection, this modulated parasite-specific response appears to extend to non-filarial (bystander) antigens including orally- and parenterally delivered vaccines (13–26). Although there have been a significant number of studies examining the immunological aspects *of L. loa, O. volvulus, W. bancrofti*, and *B. malayi* infections in humans, very few have investigated the subsets and the function of regulatory T cells in these infections. Though initial epidemiological and immune response studies were done in human populations, the majority of studies investigating the mechanisms underlying the regulation of these immune responses have been performed in animal studies. For instance, although antigen-specific T cell hypo-responsiveness in filarial infection was first described in human in *in vitro* systems, studies investigating role played by regulatory T cells have been carried out in murine models of filarial infection. Moreover, with accumulating evidence that multiple subsets of regulatory T cells exist, based on the expression of particular transcription factors, their origin and/or the regulatory cytokines they produce (27–31), animal models have been critical in understanding the function of a given subset in the context of filarial infection. Thus, the present review will focus on the different subsets of regulatory T cells in the context of chronic filarial infection (mostly *W. bancrofti* and *O. volvulus*) of humans as well as in studies using relevant animal models.

### Immune regulation in filarial infections

Early studies of immune responses in LF showed that while individuals with circulating microfilariae showed impaired filarial-specific lymphoproliferative responses and cytokine (IL-2 and IFN-γ) production, cells from individuals free of parasites and free of clinical symptoms (so-called endemic normals) and from those with lymphedema (but no circulating filarial antigenemia) proliferated vigorously and produced measurable levels of cytokines to filarial parasite antigen (6, 32–37). Because all of these earlier studies were cross-sectional and in human populations, it remained unclear how the down-regulated antigen-specific T cell response in those with patent infection got established. However, based on animal models of filarial infection (e.g., *Litomosoides* or *Brugia*) and some limited studies *in vitro* using human cells exposed to infectious stage larvae (38–42), in our opinion the majority of data point to time-dependent early response to filarial parasites in which the mammalian-adapted infective larvae (L3) induce a local inflammatory response that is followed by a mixed type 1 (Th1) and type 2 (Th2) T cell response with higher levels of IL-4 and IL-5 cytokines (43–46). At the time of patency (that is when microfilariae appear in the blood or skin) there is (again based on varying animal models with different times to patency (45, 47) – a change in the parasite-specific immune response in which a Th2-expanded immune response occurs (with a concurrent contraction of the Th1 response) that is followed by a modulated (regulated) response that is mediated by IL-10 and TGF-β (among others) (48–52).

That soluble factors and suppressive cells might mediate the immune hypo-responsiveness associated with chronic filarial infection was first suggested by work in a *B. malayi*-endemic region of Indonesia (19). Furthermore using animal models, it has been shown that the suppression of filarial-specific immune response during chronic filarial infection was mediated by non-specific suppressor cells (33). In fact, it was known since the early 1970s that T cells mediated some of the suppression of immune responses engendered in mice; by the mid 1990s regulatory T cells were identified in mice followed subsequently by their having been found in humans (53–59).

Though regulatory T cells were discovered about two decades ago, questions remain about their basic biology, their mode of action, and their therapeutic potentials. Moreover, a number of regulatory T lymphocytes (Tregs) have been described. Based on the expression of the canonical transcription factor Foxp3, two Foxp3+ subsets have been identified: the regulatory T cells (Tregs) that are thymus-derived (tTregs) and those that are induced in the periphery from naïve Foxp3-T cells or pTregs (60). In addition to the Foxp3-expressing Tregs, two other subsets that do not express Foxp3 have been described based on the regulatory cytokines expressed by those cells. These include the type 1 regulatory T cells (Tr1) that express mainly IL-10 and the TGF-β expressing Th3 regulatory T cells (27, 28, 61–65). Each of the Treg subsets has been identified in the peripheral blood of filarial-infected patients.

Following the discovery of the transcription factor forkhead box P3 (Foxp3) being a canonical marker of regulatory T cells (66, 67), work investigating the role of these T cells in the context of chronic filarial infection was undertaken. Indeed, by the use of multiparameter flow cytometry and qPCR, several studies showed that chronic filarial infection was associated with increased expression of Foxp3-expressing CD4+ cells as well as Foxp3 negative CD4+ cells that expressed IL-10 (68–70). These studies revealed that in patent filarial infection the immune environment is dominated by increased frequencies of regulatory T cells some of which being Foxp3-expressing T cells.

### Role of the cytokines IL-10 and TGF-β

Although IL-10 and TGF-β were originally thought to be produced by Th2 cells and can be produced by various cell types including regulatory T cells, it has been shown that the major sources of IL-10 and TGF-β are Tr1 and Th3 respectively (71–78). Immune responses to filarial infection have been shown to be stage-specific with cytokines such as IL-4, IL-2, IFN-γ, IL-5, and IL-13 in association with IgE dominating the acute phase of the infection while levels of regulatory cytokines such as IL-10 and TGF-β and the antibody isotype IgG4 being elevated during the chronic phase of the infection (79–83). The role of the cytokines IL-10 and TGF-β in the modulation of immune responses during patent filarial infection was largely inferred from studies demonstrating that neutralizing antibodies to IL-10 (and to a lesser extent TGF-β) significantly increased the down-regulated antigen-specific proliferative responses in patients with subclinical microfilaremic *W. bancrofti* infection (1). In similar studies in Haiti (*W. bancrofti*-endemic) data emerged to show that cells from microfilaremic subjects also showed an inverse relationship between proliferative response to filarial antigens and IL-10 production in filarial-infected individuals (84). Since these initial studies, others have extended these by demonstrating that high levels of IL-10 were produced spontaneously (*ex vivo*) and in response to parasite antigen stimulation in filarial-infected individuals (85, 86). Additional studies using neutralizing antibodies to IL-10 (as well as TGF-β) reversed both the T cell hypo-responsiveness and cytokine production to filarial antigen observed in filarial-infected patients (1, 69, 87, 88) and also reversed some of the modulation seen to the response to bystander antigens (24). The critical role of IL-10 in modulating immune responses during chronic filarial infection has been shown most notably in animal models of infection. In fact, it has been shown that mice treated with anti-IL-10 neutralizing antibody or in IL-10 deficient mice had lower microfilaremia (with *B. malayi*) compared with isotype treated or wild type mice (89).

In addition to directly suppressing immune responses IL-10 and TGF-β may indirectly regulate not only the antibody response to filarial antigens but also the function of antigen presenting cells (APCs) (1, 49, 52, 90). In fact, it has been shown that IgG4 is associated with patent filarial infection while IgE was associated with the acute phase of the infection (79, 82, 83, 91–94). Furthermore, IgE and IgG4 seem to be strongly induced in filarial infection; while IgE appears very early in the infection, IgG4 levels rises exponentially following the production of microfilaremia.

The mixed IgE/IgG4 seen in chronic filarial infection may reflect the cytokine environment that dominates the immune environment during chronic infection. In fact, it has been shown that patent filarial infection is characterized by a modified Th2 response that is associated with increased frequencies of IL-4- and IL-10-producing CD4 T cells (70, 95). Moreover, IgG4 has been used as a marker of filarial infection diagnosis but also as a marker of immunoregulation (96, 97). Although direct evidence for filarial-induced IL-10 to be involved in the induction of IgG4 class switching has not been established, it has been shown that IL-10 can act on human B cells and induce the production of IgG4 (98, 99). Furthermore, Satoguina and collaborators showed tetanus-specific regulatory T cells clone producing high levels of IL-10 and TGF-β induced the production of IgG4 by naive and memory B cells in a GITR/GITRL-, TGF-β-, and IL-10-dependent manners (100). In addition to modulating antibody responses, it has been shown that chronic filarial infection modulates the function of APCs. In fact, APCs from filaria-infected animals appear to promote T cell unresponsiveness (49, 90, 101–104).

### Regulatory T cells in filarial infection

With the identification of CD25+CD4+ T cells as a subpopulation responsible for controlling autoimmunity and for downregulating immune responses in mice (54–56, 105), these regulatory T cells (Tregs) were demonstrated in humans at relatively consistent levels in human peripheral blood (57, 58, 106). In patients with LF, it was first demonstrated that Foxp3, CTLA-4, TGF-β, and PD1 expression in bulk PBMCs were significantly increased in filaria-infected individuals (69). Concurrently, several studies in mouse models of filarial infection and in human populations showed that filarial infection was associated with increased frequencies of these Tregs (70, 107–111). Using a non-permissive mouse model of infection with *B. malayi*, it was then shown that mice infected with either infective stage larvae or implanted with adult parasites expanded a population of CD4+Foxp3+ T cells that also expressed CD25, CD103, and CTLA-4 (107). Using multicolor flow cytometry in a filarial-infected group of patients in Mali, it was further shown that human filarial infection was also associated with an increased frequency not only of Tregs that were CD4+CD25+Foxp3+CD127−, but also of CD4+CD25−Foxp3−cells producing only IL-10 [characteristic of type 1 regulatory (Tr1) cells] (70).

Several studies have reported an increased frequency of Foxp3-expressing Tregs in filarial infection in humans and in animal models (69, 70, 107, 110, 112) though the differentiation between tTregs and pTregs in peripheral blood circulation has not been addressed clearly to date (29, 31, 113, 114). Recently, using a mouse model of the intestinal helminth parasite *Heligmosomoides polygyrus*, it has been demonstrated that E/S products of this parasite contained a TGF-β-like molecule that was sufficient to induce *in vitro* the differentiation of Foxp3-expressing Tregs or iTregs (115). Although this induction of iTregs by filarial parasites has not been assessed in humans, it has been shown that infection of mice with human filarial parasite *B. malayi* or the murine filarial parasite *L. sigmodontis* induce early expression of Foxp3 and recruitment of Foxp3-expressing regulatory T cells (107, 109, 110). Furthermore, it has been shown that all filarial parasites examined to date do express a homolog of human TGF-β (116–119). Furthermore, using onchocercomas collected from patients in West Africa, immunohistochemical staining showed that dead (but not live) *Onchocerca* adult worms in these onchocercomas were surrounded by Foxp3-expressing T cells. Whether this increased frequency of Foxp3-expressing T cells was the result of increased accumulation of tTregs or a local induction of pTregs within the tissue remains to be determined (120).

Although the difference between tTregs and pTregs has not been clearly established in filarial infection, several studies using human T cell cloning and others in mouse animal models of filarial infection have investigated Tr1 and Th3 regulatory T cells in filarial infection. T cell clones from patients with onchocerciasis were shown to produce high levels of IL-10 and TGF-β in response to parasite antigen; these cells were shown to be either Tr1 (IL-10-producing) or Th3 (TGF-β producing) cells (50). Likewise cloned T cells that produced neither IL-2 nor IL-4 but substantial amounts of IL-10 (characteristics of Tr1) that inhibited the function of other T cells *in vitro* was demonstrated from patients in Ghana (121). When looked at systematically, studies in filarial-infected patients from West Africa (but evaluated in North America) demonstrated that the major T cell source of IL-10 comes from CD4+CD25− cells (that are likely Tr1 cells) (122). These data have been supported by multiparameter flow cytomtetry based frequency analysis as well (70).

## Function of Regulatory T Cell Subsets in Filarial Infection

**Figure 1:**
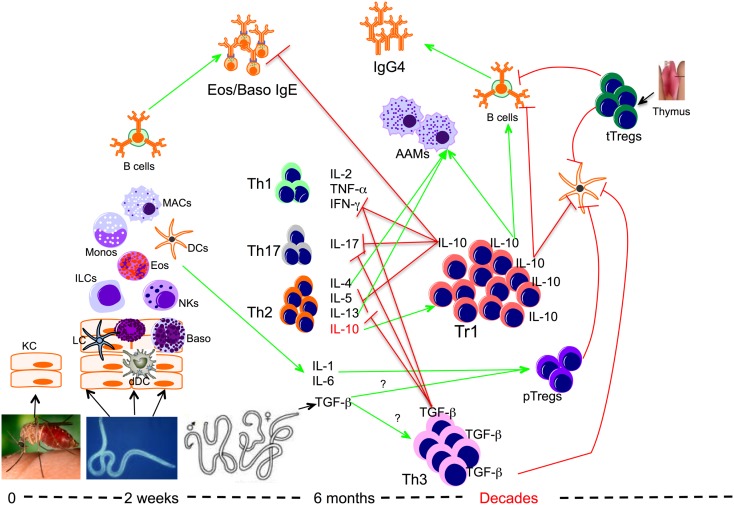
**Role of regulatory T cells in the context of filarial infection**. Filarial parasite infective larvae (L3) deposited on the skin during the bite of an infective mosquito actively penetrate the skin following which they migrate to a draining lymph node. During their migration, L3 contacts and activates different cells such as keratinocytes (KC), dermal dendritic cells (dDC), innate lymphoid cells (ILCs), macrophages (MAC), dendritic cells (DCs), and basophils (Baso). At this relatively early phase of infection the parasite induces the differentiation of effector Th1, Th17, and Th2 cells, which together with IgE antibody may lead to attrition of some of the parasites. However if there is failure to clear the parasites, the infection evolves into a chronic longstanding infection associated with IL-10-producing type 1 (Tr1), TGF-β-producing Th3, and Foxp3-expressing Tregs or peripheral Tregs (pTregs), which together with the thymus-derived Tregs (tTregs) can be found with increasing frequencies in filarial infections. The high levels of IL-10 produced induce the production of IgG4 and together with IL-4, IL-13, and/or TGF-β induce the differentiation of alternatively activated macrophages (AAM) and inhibit the function of a variety of other cells.

Several mechanisms by which Tregs (tTregs/pTregs, Tr1, and Th3) mediate their suppressive functions have been investigated in the settings of chronic filarial infection (Figure 1). Though their mode of action is not very clear, it is thought that tTregs and pTregs (at least) mediate their suppressive function through cell to cell interaction through surface molecules such as CTLA-4, GITR, LAG-3, and membrane-bound TGF-β (123–127). In chronic filarial infection settings studies investigating the mechanisms underlying the immune hypo-responsiveness showed that CD4+ cells from filaria-infected individuals not only expressed high levels of CTLA-4 but that antibody blockade of CTLA-4 in *in vitro* cultures increased filarial antigen-specific proliferative response and cytokine production (87). Likewise, it has been shown that antibody blockade of CTLA-4 and TGF-β *in vitro*, increased the expression of IFN-γ, TNF-α, IL-4, IL-5, GATA-3, and Tbet messenger RNA by cells from filaria-infected subjects in response to parasite antigen stimulation (69).

*In vivo* depletion of regulatory T cells using anti-CD25 and antibody in combination with anti-GITR antibody in a mouse model of filarial infection demonstrated enhanced production of IL-4, IL-5, and IL-10 in response to parasite antigen stimulation *in vitro* (109). In addition these authors showed that neutralization of CTLA-4 and depletion of CD4+CD25 regulatory T cells in combination increased parasite-specific antibody production and enhanced worm killing (108).

Though the direct effect of filaria-induced Tregs on APC has not been evaluated formally, several studies have shown that APCs from those with patent filarial infection have altered phenotypes and diminished function (49, 90, 101, 103, 104, 128–133). Although the mechanisms underlying the modulation of APC function in patent filarial remain obscure, several studies suggested that the regulatory cytokines TGF-β and IL-10 might involved. Furthermore it has been shown that tTregs and/or pTregs modulate APC function through molecules such as CTLA-4, GITR, LAG-3, and membrane-bound TGF-β (123–127).

Though the role of tTregs and pTregs in the context of human filarial infection remains elusive, the other regulatory T cells subsets act thought the production and secretion of IL-10 and TGF-β (1, 69, 87, 88). Although these regulatory cytokines can be produced by different types of CD4 T cells including tTregs and pTregs, in the setting of filarial infection, it has been showed that the principal sources of IL-10 and TGF-β are Tr1 and Th3 cells respectively (50, 70, 121, 122). Using animal models of filarial infection it has been shown that these regulatory cytokines particularly IL-10 directly regulate immune response to filarial parasites (89, 134). These regulatory cytokines elevated in the serum of chronically infected individuals and together with Foxp3-expressing surface markers have been shown to also modulate in these individuals immune responses to non-filarial antigens including malarial antigens (24, 25, 135–138), mycobacterial antigens (139), and antigens associated with type 1 diabetes (140, 141).

## Conclusion

Despite the rapidly accumulating evidence acknowledging the existence of multiple subsets of Tregs and their general modulation of immune responses, the understanding of the molecular mechanisms of their mode of action is still limited. What is clear in chronic filarial infection is an association of infection with increases of most of the Tregs subsets; however it is the dominance of IL-10-mediated regulation that seems to be the most consistent finding suggesting that the Tr1 cells (along with conventional IL-10-producing Th2 cells) play the major role.

Delineating the subsets and function of Tregs is of capital importance as this would provide insight into their model of action and enhance their use as potential therapeutic targets. Despite recent advances in the understanding of Treg functions the lack of simple surface expressed markers for each subset has hindered some of the fundamental research on their mechanisms of action. Despite this lack of mechanistic insight, these regulatory T cells are clearly responsible for the modulation of parasite antigen-specific responses so characteristic of patent filarial infections.

## Conflict of Interest Statement

Because Simon Metenou and Thomas B. Nutman are government employees and this is a government work, the work is in the public domain in the United States. Notwithstanding any other agreements, the NIH reserves the right to provide the work to PubMedCentral for display and use by the public, and PubMedCentral may tag or modify the work consistent with its customary practices. You can establish rights outside of the U.S. subject to a government use license.
